# Biomarker-based prediction of radial artery occlusion after cardiac catheterization

**DOI:** 10.1590/1414-431X2025e15093

**Published:** 2026-03-27

**Authors:** C. Alp, H. Kandemir, S. Ozturk

**Affiliations:** 1 Department of Cardiology, Kırıkkale University Faculty of Medicine, Kırıkkale, Turkey

**Keywords:** Creatinine, Estimated glomerular filtration rate, Platelet, Radial artery occlusion, Transradial angiography

## Abstract

Thrombotic occlusion of the radial artery is the most frequent complication of transradial angiography. This study sought to investigate biomarker-based predictors of radial artery occlusion (RAO) after coronary angiography (CAG) and/or percutaneous coronary intervention (PCI). Consecutive patients who underwent cardiac catheterization through radial artery route for diagnostic or therapeutic purposes were included in the study retrospectively. The clinical, laboratory, and angiographic data were obtained from hospital records. All patients were invited for a follow-up visit one week after discharge and radial artery pulse examination was performed. The patients with reduced or absent radial artery pulse or complaints related with the radial artery intervention site at follow-up visit were examined by a radiologist with superficial Doppler ultrasonography. The patients were categorized according to the patency of the radial artery and there were 46 patients with an occluded radial artery and 204 patients with a patent radial artery after CAG and/or PCI. Platelet count was higher in the occluded artery group than in the patent radial artery group. Creatinine level was lower and estimated glomerular filtration rate (e-GFR) was higher in the occluded radial artery group compared to the patent radial artery group. Multivariate logistic regression analysis showed that platelet count (OR: 1.010, 95%CI: 1.001-1.018, P=0.031) and creatinine (OR:0.030, 95%CI: 0.001-0.821, P=0.038), but not e-GFR (OR: 1.031, 95%CI: 0.991-1.073, P=0.128) were independently associated with RAO. Platelet count and creatinine were found to be independent predictors of RAO after transradial cardiac catheterization.

## Introduction

Cardiac catheterization procedures such as coronary angiography (CAG) and percutaneous coronary intervention (PCI) are being performed more frequently through radial artery cannulation in the modern interventional cardiology era (1). Radial artery access has many advantages over the transfemoral route such as rapid hemostasis, decreased bleeding complications, improved patient safety, earlier patient mobilization, greater patient comfort, and decreased hospital stay and cost (2). Recent studies comparing radial and femoral artery routes for CAG and PCI also indicate improvements in cardiovascular events, including lower mortality, in favor of the radial artery access (3,4). Despite the advantages of transradial access, complications such as forearm hematoma, dissection, pseudoaneurysm, spasm, arteriovenous fistula, and thrombotic occlusion of the radial artery may occur during or after the procedure (5). Patency of the radial artery is important due to the possibility of future interventions, the need for coronary artery bypass graft, and/or use of a hemodialysis conduit (2).

Thrombotic occlusion of the radial artery is the most frequent complication of transradial angiography. The reported incidence of radial artery occlusion (RAO) after intervention varies from 0.8 to 38% in the literature (2,6). The clinical presentation might vary from asymptomatic occlusion to critical hand ischemia depending on the vascular supply of the hand from the palmar arch. A number of clinical and procedural factors such as body mass index (BMI), diabetes mellitus (DM), sheath size, anticoagulation status and patent hemostasis have been shown to be associated with RAO after transradial interventions (2). On the other hand, there is an interest and paucity of data for biomarker-based prediction of RAO after CAG and PCI. C-reactive protein (CRP)-to-albumin ratio, a marker of systemic inflammation, was recently defined as an independent predictor of radial artery thrombosis (RAT) after transradial angiography (7). Another study found increased platelet count, platelet distribution width, and high-sensitivity CRP (hs-CRP) as independent predictors of RAO after cardiac catheterization (8). In a similar manner, our study sought to investigate biomarker-based predictors of RAO after CAG and/or PCI.

## Material and Methods

### Study design and population

Consecutive patients who underwent cardiac catheterization through the radial artery route for diagnostic or therapeutic purposes at our clinic between July 2024 and January 2025 were retrospectively included in the study. The clinical, laboratory, and angiographic data were obtained from hospital records. During the defined time frame, 1225 patients underwent a cardiac procedure at the catheterization laboratory. The patients who underwent catheterization through femoral artery cannulation, any cardiac procedure other than CAG and/or PCI, previously used the same radial route, and/or with missing data were excluded from the study. After the application of exclusion criteria, 250 patients remained and were included in the statistical analysis. All of the study procedures were conducted in line with the Recommendations of the Good Clinical Practices Guidelines and the Declaration of Helsinki. An informed consent was not obtained from the patients. The study protocol was approved by the Ethics Committee of the University hospital (2025.04.21).

Individuals were considered to have arterial hypertension (HTN) if they had a previously documented history of HTN, with or without antihypertensive therapy, or a mean office blood pressure repeatedly measuring ≥140/90 mmHg. The diagnosis of DM was based on current use of glucose-lowering medications, fasting glucose levels equal to or above 126 mg/dL, or non-fasting glucose levels of 200 mg/dL or higher. A diagnosis of coronary artery disease (CAD) was established based on a documented history of myocardial infarction (MI), previous coronary revascularization, or angiographic evidence of plaque formation and/or arterial obstruction. The patients with an estimated glomerular filtration rate (e-GFR) below 90 mL/min/1.73 m^2^ lasting at least three months were diagnosed as having chronic kidney disease (CKD).

### Laboratory data

Blood samples of the patients were obtained from an antecubital vein and collected in standardized EDTA-containing tubes for complete blood count test. All blood samples were processed within 30 min after collection and analyzed in the hospital’s biochemistry laboratory. Fasting blood samples were used for lipid panel and glucose measurements. Alanine aminotransferase (ALT), aspartate aminotransferase (AST), creatinine, glucose, serum electrolytes including sodium and potassium, thyroid-stimulating hormone (TSH), and lipid panel were measured using standard methods. Serum CRP levels were determined by the turbidimetric method. e-GFR was calculated according to the formula developed in 2009 by the Chronic Kidney Disease Epidemiology Collaboration (9). The study used results of routine protocol-based laboratory evaluations performed as part of standard patient care.

### Cardiac catheterization and follow-up

The left radial artery was cannulated with a 6 French (F) or 7F sheath through the Seldinger technique in all patients who had a palpable radial pulse. First, local subcutaneous anesthesia with 2 cc of 1% prilocaine was administered to the intervention region while the left hand was in extension and external rotation. Afterwards, radial artery puncture was performed with a specialized cannulation needle and guidewire. Following the insertion of the sheath, 200 mcg of nitroglycerin and 5000 IU of unfractionated heparin was applied in order to prevent radial artery spasm and thrombosis. Diagnostic CAG was performed using 5F or 6F catheters. An additional unfractionated heparin dose of 100 IU/kg was applied if a decision was made to proceed with PCI after CAG. The decision for continuation of heparin and/or glycoprotein IIb/IIIa inhibitor usage was at the discretion of the operators. PCI procedures were performed using 6F or 7F catheters. All procedures were performed by experienced operators. Catheter selection was based on coronary anatomy, catheterization availability in the laboratory, and operator discretion. The radial sheath was removed immediately at the end of the procedure, and hemostasis was achieved using the patent hemostasis technique via a hemostatic compression device (BIOHENGE^®^ Transradial Compression Device, Turkey) inflated to the puncture site with 15-20 mL of air, which was removed after 2 h.

All patients underwent two-dimensional transthoracic echocardiography before or after the cardiac catheterization depending on the clinical presentation. Echocardiographic examination was performed at lateral decubitus position by using the General Electric VIVID E9 (USA) device. The in-hospital and discharge pharmacological treatment of the patients was compatible with the European Society of Cardiology guideline recommendations (10,11). All patients were invited for a follow-up visit one week after discharge and radial artery pulse was examined. Patients with reduced or absent radial artery pulse or complaints related to the radial artery intervention site were examined by a radiologist with superficial Doppler ultrasonography and RAO was determined.

### Statistical analysis

Statistical analyses were performed using SPSS 22.0 (Version 22.0, IBM Corp., USA). Data normality of the continuous variables was assessed by the one-sample Kolmogorov-Smirnov test and visual methods including histograms and probability plots. Normally distributed continuous parameters are reported as means±SD and group comparisons were made by the independent-samples *t*-test. Non-normally distributed continuous parameters are reported as median and 25th and 75th percentiles and compared with Mann-Whitney-U test. Chi-squared test or Fisher exact test was used for comparison of categorical variables.

Logistic regression analysis was carried out to examine biomarker-based predictors of RAO, and the results are reported as odds ratio (OR) and 95% confidence interval (CI). Blood parameters that demonstrated statistical significance in comparative analyses were first tested with univariate regression analysis. Parameters with a P value <0.1 in the univariate analysis were included in the multivariate logistic regression analysis. Each blood parameter was examined in separate multivariate models in order to avoid multicollinearity and model overfitting, and adjustments were performed subsequently. Adjusted parameters were chosen among clinical variables that demonstrated statistical significance in comparative analyses and could be associated with RAO such as age, gender, CAD, left ventricular ejection fraction, presentation type, treatment of choice, beta blocker prescription at discharge, statin prescription at discharge, and P_2_Y_12_ inhibitor prescription at discharge. The multicollinearity assessment was performed by the r correlation coefficient. The variables that yielded a correlation coefficient value ≥0.7 were considered to have multicollinearity and were not examined in the same model. The goodness-of-fit was tested by the Hosmer-Lemeshow method for each model and considered satisfactory when P >0.05.

The receiver operating characteristic (ROC) curve analysis was performed to determine the cut-off values for the sensitivity and specificity of platelet count in predicting RAO and the creatinine level in predicting patent radial artery. The area under the ROC curve (AUC) was represented with 95%CI, sensitivity, and specificity. A two-sided P value <0.05 was defined as statistically significant for all tests.

## Results

The overall study population consisted of 250 patients. The mean age of the study group was 58.73±11.90 years and 45.6% of the patients were female. The overall frequency of HTN, DM, CAD, and CKD was 53.2, 26.8, 30.0, and 49.2%, respectively. The presentation type was acute coronary syndrome (ACS) in 29.2% of the patients and 34% of the study population was treated with PCI. The patients were categorized according to radial artery patency: 46 patients had an occluded radial artery and 204 patients had a patent radial artery at follow-up outpatient visit after catheterization (Table 1).

**Table 1:** Baseline demographic characteristics and medications of the study groups.

Variables	Overall study group (n=250)	Occluded radial artery (n=46)	Patent radial artery (n=204)	P value
Age, years	58.73±11.90	53.89±9.77	59.82±12.08	**0.002**
Gender (female)	114 (45.6)	28 (60.9)	86 (42.2)	**0.021**
Active smoking	52 (20.8)	12 (26.1)	40 (19.6)	0.328
Hypertension	133 (53.2)	22 (47.8)	111 (54.4)	0.419
Diabetes mellitus	67 (26.8)	11 (23.9)	56 (27.5)	0.625
Hyperlipidemia	39 (15.6)	5 (10.9)	34 (16.7)	0.328
Coronary artery disease	75 (30)	8 (17.4)	67 (32.8)	**0.039**
Chronic kidney disease	123 (49.2)	15 (32.6)	108 (52.9)	**0.013**
Respiratory disease	9 (3.6)	0 (0)	9 (4.4)	0.147
Atrial fibrillation	15 (6)	0 (0)	15 (7.4)	0.058
Anemia	57 (22.8%)	14 (30.4%)	43 (21.1%)	0.189
LVEF (%)	58 (56-61)	60 (58-64)	58 (55-60)	**<0.001**
Presentation type, ACS	73 (29.2)	4 (8.7)	69 (33.8)	**0.001**
Treatment of choice, PCI	85 (34)	9 (19.6)	76 (37.3)	**0.027**
Baseline medications				
Acetylsalicylic acid	70 (28)	9 (19.6)	61 (29.9)	0.158
P_2_Y_12_ inhibitor	26 (10.4)	2 (4.3)	24 (11.8)	0.137
Oral anticoagulant	5 (2)	0	5 (2.5)	0.283
Statin	46 (18.4)	5 (10.9)	41 (20.1)	0.145
Beta blocker	60 (24)	7 (15.2)	53 (26)	0.123
RAAS inhibitors	69 (27.6)	14 (30.4)	55 (27)	0.634
Calcium channel blocker	33 (13.2)	4 (8.7)	29 (14.2)	0.318
Diuretic	41 (16.4)	7 (15.2)	34 (16.7)	0.810
Antidiabetic	47 (18.8)	8 (17.4)	39 (19.1)	0.787
Nitrate	3 (1.2)	0	3 (1.5)	0.408
Discharge medications				
Acetylsalicylic acid	196 (78.4)	40 (87)	156 (76.5)	0.118
P_2_Y_12_ inhibitor	88 (39.6)	8 (19)	80 (44.4)	**0.002**
Oral anticoagulant	14 (6.3)	0	14 (7.8)	0.078
Statin	146 (65.8)	22 (52.4)	124 (68.9)	**0.042**
Beta blocker	129 (58.1)	13 (31)	116 (64.4)	**<0.001**
RAAS inhibitors	81 (36.5)	10 (23.8)	71 (39.4)	0.058
Calcium channel blocker	42 (18.9)	6 (14.3)	36 (20)	0.395
Ranolazine	14 (6.3)	1 (2.4)	13 (7.2)	0.478
Diuretic	53 (23.9)	2 (4.8)	51 (28.3)	**0.001**
Nitrate	8 (3.6)	0	8 (4.4)	0.358

Data are reported as means±SD, median (25th-75th percentile), or number (%). P-values in bold indicate statistical significance. Independent-samples *t*-test, Mann-Whitney-U test, chi-squared test, or Fisher exact test. ACS: acute coronary syndrome; LVEF: left ventricle ejection fraction; PCI: percutaneous coronary intervention; RAAS: renin angiotensin aldosterone system.

The patients in the occluded radial artery group were more likely to be younger and female (P=0.002, P=0.021, respectively). The frequency of active smoking, HTN, DM, hyperlipidemia, respiratory disease, atrial fibrillation, and anemia was comparable between groups, whereas CAD and CKD were less common in patients with occluded radial artery than patent radial artery (P=0.039, P=0.013, respectively). The left ventricular ejection fraction median was higher in the occluded radial artery group (P<0.001). ACS presentation and treatment with PCI were less common in the occluded radial artery group (P=0.001, P=0.027, respectively). There was no difference between groups in terms of baseline medications, whereas patients with occluded radial artery were less likely to be discharged with a P_2_Y_12_ inhibitor, statin, beta blocker, and diuretic therapy (P=0.002, P=0.042, P<0.001, P=0.001, respectively) (Table 1).

There was no difference among groups in terms of hemoglobin, neutrophil, and monocyte counts. Lymphocyte count (2.88 (2.23-3.39) ×10^9^/L, 2.14 (1.63-2.65) ×10^9^/L, P=0.004, respectively) and platelet count (321 (269-377.5) ×10^9^/L, 225 (184-263×10^9^/) ×10^9^/L, P<0.001, respectively) were higher in the occluded radial artery group than in the patent radial artery group. The median ALT was similar among groups, but AST tended to be lower in the occluded radial artery patient group (P=0.036). There was no difference between groups regarding sodium, potassium, glucose, CRP, and TSH levels, but creatinine (0.74 (0.58-0.82) mg/dL, 0.85 (0.73-1.01) mg/dL, P<0.001, respectively) level was lower and e-GFR (95.91±13.5 mL/min/1.73 m^2^, 82.34±18.84 mL/min/1.73 m^2^, P<0.001, respectively) was higher in the occluded radial artery group compared to the patent radial artery group. There was no difference among groups regarding the lipid panel (Table 2).

**Table 2:** Laboratory findings of the study groups.

Variables	Occluded radial artery (n=46)	Patent radial artery (n=204)	P value
Hemoglobin (g/L)	13.7±2.0	13.88±1.85	0.713
Neutrophils (×10^9^/L )	4.73±0.95	5.1±1.77	0.731
Monocytes (×10^9^/L)	0.52 (0.36-0.63)	0.47 (0.37-0.62)	0.784
Lymphocytes (×10^9^/L )	2.88 (2.23-3.39)	2.14 (1.63-2.65)	**0.004**
Platelets (×10^9^/L)	321 (269-377.5)	225 (184-263)	**<0.001**
Alanine amino transferase (U/L)	18.5 (13-37)	15 (12-20)	0.876
Aspartate amino transferase (U/L)	18 (14.5-22)	19 (16-22)	**0.036**
Sodium (mmol/L)	140.25±1.81	140.23±2.53	0.664
Potassium (mmol/L)	4.58±0.3	4.38±0.45	0.208
Creatinine (mg/dL)	0.74 (0.58-0.82)	0.85 (0.73-1.01)	**<0.001**
e-GFR (mL/min/1.73 m^2^)	95.91±13.5	82.34±18.84	**<0.001**
Glucose (mg/dL)	101 (89.5-126.5)	102 (92-129)	0.865
C-reactive protein (mg/L)	2.44 (1.1-4.05)	3.8 (1.59-6.1)	0.308
TSH (uIU/mL)	1.96 (1.3-3.29)	1.61 (1.07-2.17)	0.456
Total cholesterol (mg/dL)	181 (165.5-204.5)	181 (153-220)	0.100
Triglycerides (mg/dL)	126.5 (89.5-201)	125 (90-183)	0.674
HDL (mg/dL)	51.58±15.19	42.29±9.89	0.078
LDL (mg/dL)	99.4 (88.4-120)	109 (86.4-138)	0.506
Non-HDL (mg/dL)	126.5 (117-160.5)	137 (114-173)	0.271

Data are reported as means±SD or median (25th-75th percentile). P-values in bold indicate statistical significance. Independent-samples *t*-test or Mann-Whitney-U test. e-GFR: estimated glomerular filtration rate; HDL: high-density lipoprotein; LDL: low-density lipoprotein; TSH: thyroid stimulating hormone.

Univariate logistic regression analysis revealed a significant association of platelet count, creatinine, and e-GFR with RAO. In multivariate logistic regression analysis, platelet count (OR: 1.010, 95%CI: 1.001-1.018, P=0.031) and creatinine (OR: 0.030, 95%CI: 0.001-0.821, P=0.038), but not e-GFR (OR: 1.031, 95%CI: 0.991-1.073, P=0.128) were independently associated with RAO (Table 3).

**Table 3:** Prediction of radial artery occlusion after cardiac catheterization by logistic regression analysis.

Variables	Univariate		Multivariate*	
	OR (95%CI)	P value	OR (95%CI)	P value
Lymphocytes	1.19 (0.94-1.50)	0.139	-	-
Platelets	1.007 (1.003-1.011)	**0.001**	1.010 (1.001-1.018)	**0.031**
Aspartate aminotransferase	0.950 (0.893-1.011)	0.107	-	-
Creatinine	0.013 (0.002-0.104)	**<0.001**	0.030 (0.001-0.821)	**0.038**
e-GFR	1.039 (1.017-1.060)	**<0.001**	1.031 (0.991-1.073)	0.128

*Adjusted for age, gender, coronary artery disease, LVEF, presentation type, treatment of choice, beta blocker prescription at discharge, statin prescription at discharge, and P_2_Y_12_ inhibitor prescription at discharge. Each independent variable was evaluated in separate models and variables that correlated significantly among themselves were not included in the same model in order to avoid multicollinearity and model overfitting. CI: confidence interval; e-GFR: estimated glomerular filtration rate; LVEF: left ventricle ejection fraction; OR: odds ratio. P-values in bold indicate statistical significance.

**Figure 1:**
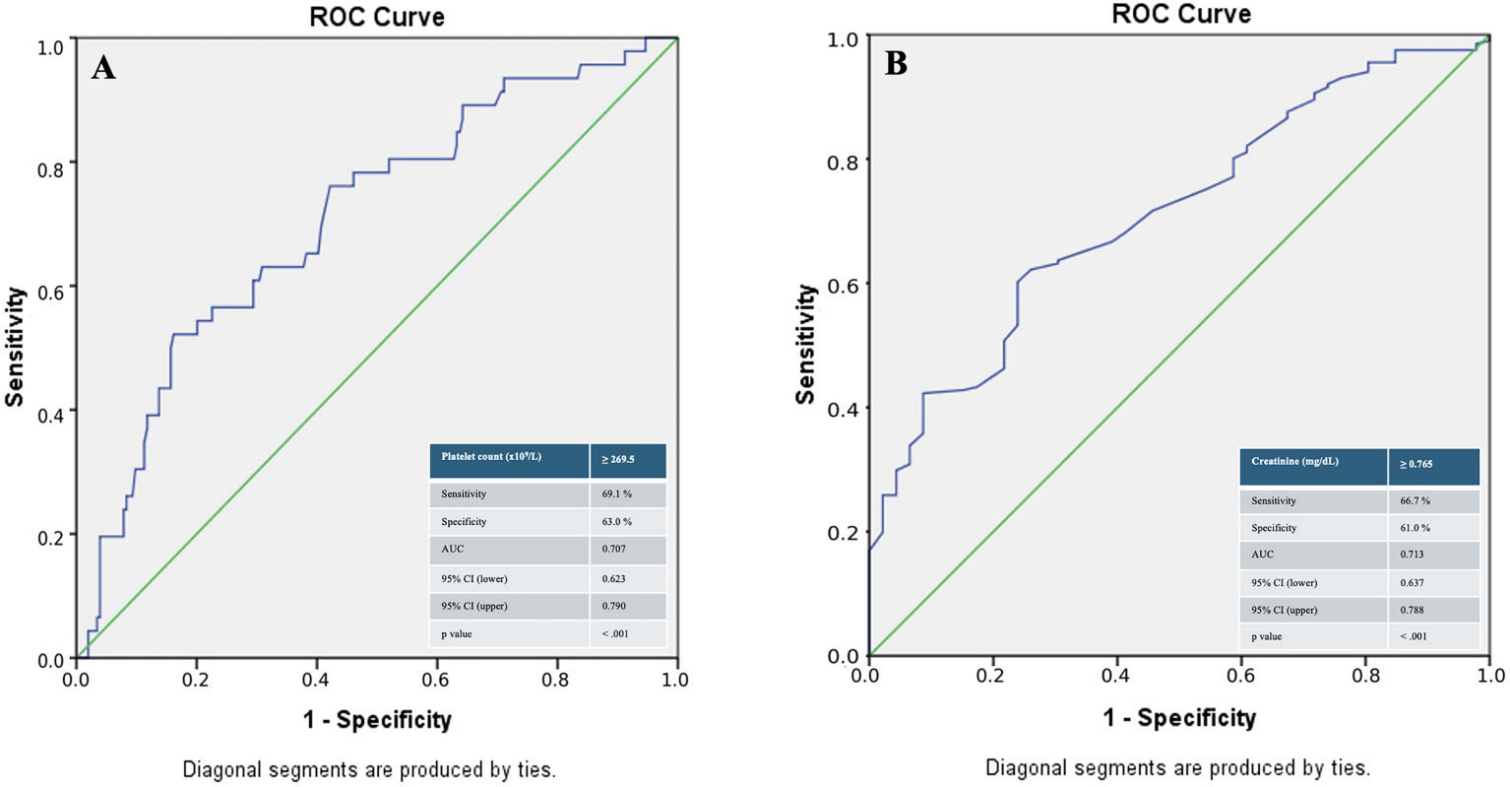
**A**, ROC curve analysis for platelet count in predicting radial artery occlusion. **B**, ROC curve analysis for creatinine in predicting patent radial artery. AUC: area under the curve, CI: confidence interval, ROC: receiver operating characteristic.

The ROC curve analysis for platelet count to predict RAO demonstrated an AUC value 0.707 (95%CI: 0.623-0.790, P<0.001). The cut-off value of platelet count (269.5×10^9^/L) yielded 69.1% sensitivity and 63.0% specificity (Figure 1A). The ROC curve analysis for creatinine to predict patent radial artery demonstrated an AUC value 0.713 (95%CI: 0.637-0.788, P<0.001). The cut-off value of creatinine (0.765 mg/dL) yielded 66.7% sensitivity and 61.0% specificity (Figure 1B).

## Discussion

Our study results indicated an independent association between RAO after cardiac catheterization and platelet count and creatinine. In contrast to the already existing literature, we did not find evidence of association between inflammation and RAO.

Platelets have substantial biological roles in the hemostatic and thrombotic processes of the vascular endothelium. They ensure the integrity of blood vessels by sealing off a damaged endothelial cell layer. On the other hand, dysregulated activation of platelets can give rise to thrombosis such as MI. Platelets also take part in the pathogenesis of atherosclerosis and vascular inflammation, which might be a trigger for thrombosis (12,13). Therefore, it is not surprising that there is an association between increased platelet count and thrombotic diseases. However, literature data concerning this issue is controversial. For instance, an epidemiological study found an association between high platelet counts and arterial thrombosis in the brain but not arterial thrombosis in the heart and venous system (14).

The pathophysiology of RAO after cardiac catheterization is multifactorial and complex and might differ from thrombosis originating from a vascular disease. Significant injury occurs in the radial artery wall after transradial angiography as demonstrated by histological and imaging studies, which results in endothelial cell dysfunction (15). For example, immediate imaging of the radial artery through optical coherence tomography after transradial angiography demonstrated intimal tears and medial dissections, and a repeated transradial procedure was the only independent predictor of chronic intimal thickening of the radial artery (16). Acute structural alterations of the radial artery caused by catheter-mediated endothelial damage together with local hypercoagulation and decreased blood flow due to compression of the artery for hemostasis might be the primary mechanisms for RAO after transradial intervention in the acute period. Repetitive interventions and catheter manipulations that lead to repeated endothelial injury and vessel remodeling can also induce thrombosis (15,16).

In line with the recently published literature, our study demonstrated an independent association between RAO and increased platelet count. Inci et al. (8) showed that increased platelet count is independently associated with RAO after cardiac catheterization. They also found that hs-CRP, a well-defined inflammation marker, is an independent predictor of RAO. Bulguroglu et al. (7) showed that CRP-to-albumin ratio, a novel marker of systemic inflammation, is an independent predictor of RAT after transradial angiography. In their study, platelet count was non-significantly higher in patients who developed RAT. In contrast to these studies, CRP level was similar in occluded and patent radial artery groups in our study. Moreover, there was no difference among groups in terms of inflammation-related biomarkers in blood count test components such as neutrophil-to-lymphocyte ratio, systemic immune inflammation index, etc. (data not shown). The reason why we did not find evidence of inflammation compared to the aforementioned studies may be due to differences in study design and patient characteristics. For instance, Bulguroglu et al. (7) included patients with chronic coronary syndrome, and Inci et al. (8) excluded acute MI patients. In addition, both studies excluded patients with renal failure (7,8). In our study, neither ACS nor renal failure was an exclusion criterion.

The independent association between platelet count and RAO after cardiac catheterization in our study raises the question whether this situation can be treated and/or prevented by antiplatelet and/or anticoagulant agents. In the study, the frequency of RAO was significantly lower in those presenting with ACS and those treated with PCI who needed additional anticoagulant and P_2_Y_12_ inhibitor therapy. A recent study found a protective role of low-dose acetylsalicylic acid from RAO in patients who underwent elective CAG (17). Additionally, combination of low-dose acetylsalicylic acid and clopidogrel, a P_2_Y_12_ inhibitor agent, yielded fewer RAO and higher self-recanalization rates compared to single antiplatelet therapy with low dose acetylsalicylic acid (18). In line with the recent literature, our data highlight the need for future studies about this topic.

Intriguingly, this study demonstrated a lower probability of RAO with declining renal function. Although CKD is commonly associated with increased cardiovascular and thrombotic risk because of a proinflammatory and procoagulant milieu, several mechanisms may paradoxically contribute to a lower incidence of RAO following transradial intervention in this patient population. One of the possible mechanisms is uremia-associated platelet dysfunction, a hallmark of advanced CKD, which impairs platelet aggregation, adhesion, and secretion due to the accumulation of circulating toxins (19). In addition, chronic anemia commonly observed in CKD reduces blood viscosity and shear stress, potentially limiting shear-induced platelet activation at the access site (20). Endothelial hyporeactivity in the radial artery, along with arterial medial calcification and vascular stiffness, may further reduce vasospasm and local thrombus formation (21). Moreover, CKD patients are frequently treated with long-term antiplatelet therapy for cardiovascular comorbidities (22), which may likely contribute to reduced RAO risk after the procedure in the study. Taken together, these factors suggest that the altered hemostatic balance and vascular changes in CKD patients, while detrimental in systemic contexts, might have conferred local protective effects against access-site occlusion after transradial angiography. Similar to our study, a recent study published by Ay et al. (23) found better renal function in patients with RAT, and creatinine levels independently predicted RAT after CAG. Additionally, although e-GFR was associated with RAO in univariate analysis in our study, this association did not remain significant after adjusting for other risk factors in the multivariate logistic regression analysis.

The frequency of RAO in the present study was approximately 20%, which is higher than the rates reported in recently published prospectively designed studies reflecting contemporary interventional cardiology practice (24,25). Didagelos et al. (24) reported a 9.5% incidence of RAO in an unselected, all-comers European population undergoing transradial coronary catheterization for diagnostic angiography and/or PCI. In the proRadial trial, Schlosser et al. (25) reported an incidence of RAO below 5%. The higher rates of RAO observed in our study might be attributable to several factors, including differences in study design, patient characteristics, procedural techniques, sheath size, and operator experience. However, every effort should be made to minimize and prevent the occurrence of RAO.

The study was subject to the inherent limitations associated with retrospective study designs such as selection bias, information bias, and limited control of confounding factors. For example, data of parameters such as BMI, radial artery diameter, procedure time, and hemostasis time would enhance our study. Furthermore, we did not evaluate all patients with Doppler ultrasonography to detect RAO. However, patients with no complaints or with patent radial pulse are not referred for radiological examination in real-world practice. As the study did not include a comparative assessment of RAO prevention methods, the findings should be interpreted with caution. Additionally, the fact that the analyses were not predefined introduced further uncertainty and may have increased the likelihood of type II (β) error.

In conclusion, platelet count and creatinine were found to be independent predictors of RAO after transradial cardiac catheterization.

## Data Availability

The data used to support the findings of this study are available from the corresponding author upon reasonable request.
